# Supplementary figures and images for: A Unique HMG-Box Domain of Mouse Maelstrom Binds Structured RNA but Not Double Stranded DNA
Source: PLoS One. 2015 Mar 25;10(3):e0120268. doi: 10.1371/journal.pone.0120268 (PMC4373776; doi:10.1371/journal.pone.0120268)

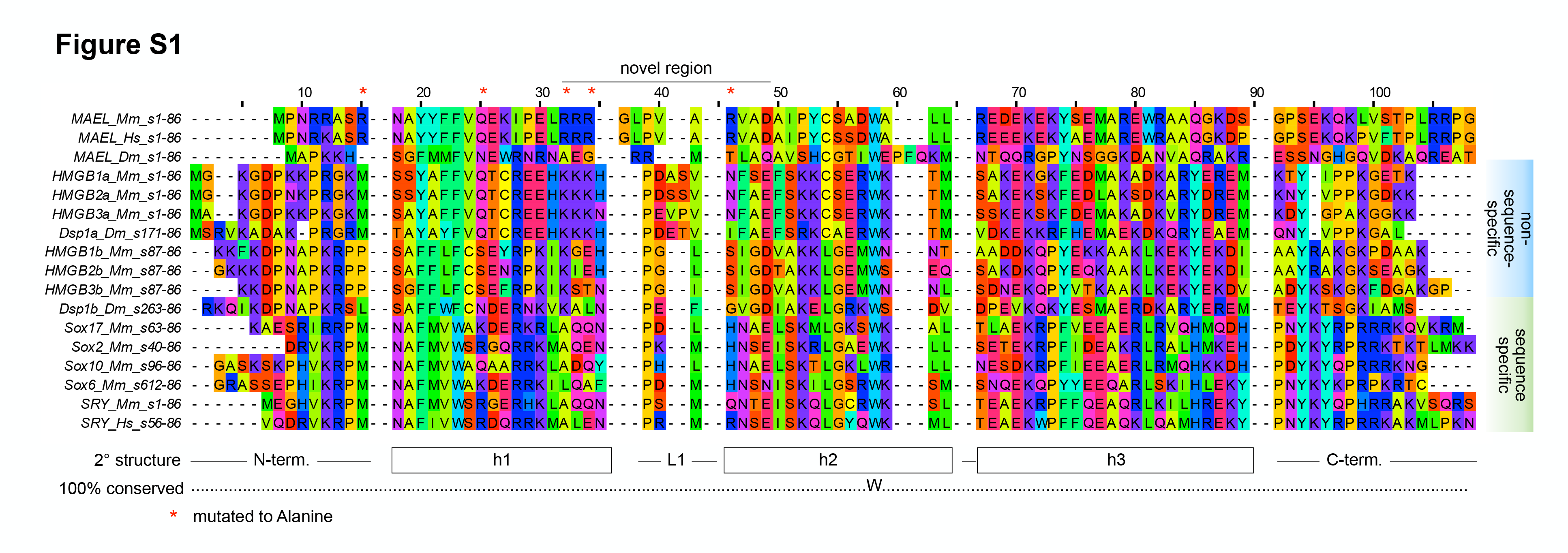

Supplement: S1 Fig — An amino acid multiple sequence alignment of candidate HMG-box domains. The candidates were selected based on their substrate specificity (sequence vs. non-sequence specific) with at least four candidates per group, and with preferences for well-described and mouse HMG-box domains. Aligned codons (ClustalW) were translated to protein sequences (MEGA6), and then the alignment was adjusted to account for the secondary structural characteristics found in solved structures within each group (SRY HMG-box-1j46, HMGB1a-1ckt, MAEL HMG-box-2cto). The residues were pseudo-colored according to Taylor color scheme (JalView) to provide contrast to groups of residues. Conserved secondary structure characteristics and residues are shown below the alignment. (TIF) [file pone.0120268.s001.tif]

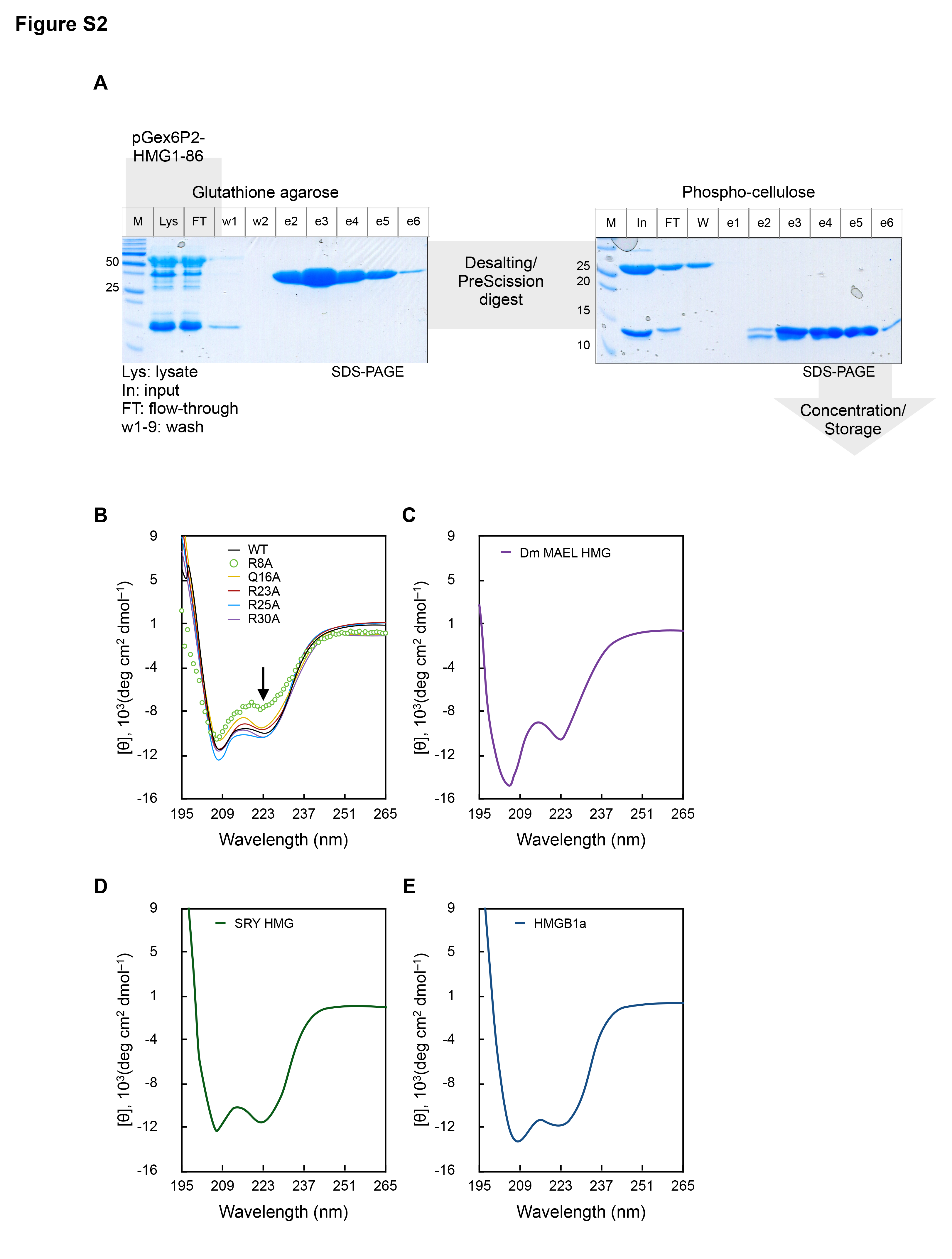

Supplement: S2 Fig — (A) Purification scheme employed for all recombinant HMG-box domain proteins. Shown are example gels demonstrating purity of acquired proteins. (B) Circular dichroism (CD) measurements of recombinant wild-type and point-mutant mouse MAEL HMG box domain proteins. All proteins are folded and helical. Only mutant R8A shows change in ellipticity (arrow) indicating that this mutation somehow affects folding of the protein. CD traces of C) Drosophila MAEL HMG-box, D) SRY HMG-box, and E) HMGB1a domains. (TIF) [file pone.0120268.s002.tif]

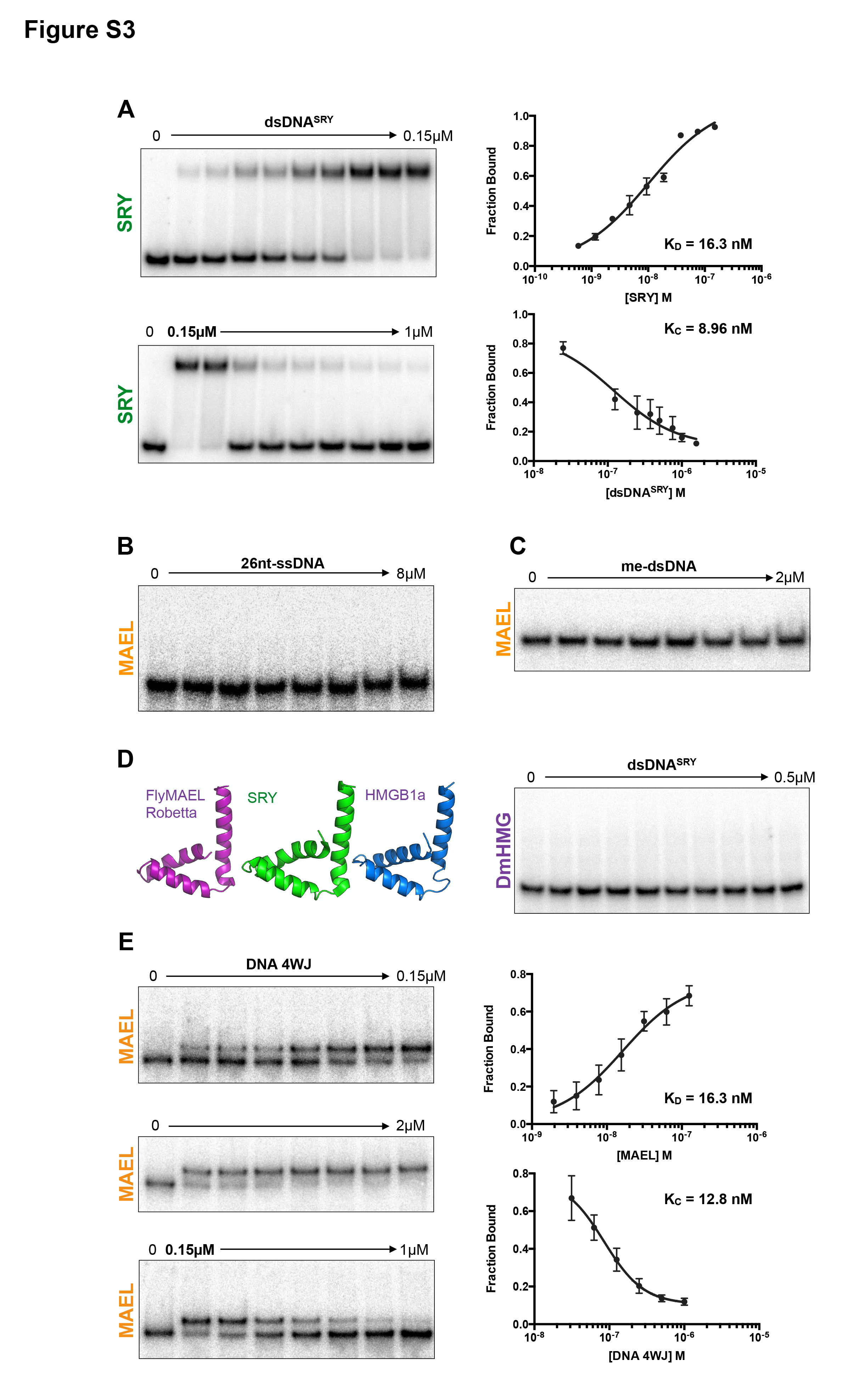

Supplement: S3 Fig — (A) Binding of SRY HMG-box domain to dsDNA with its consensus recognition sequence (dsDNASRY). Top gel shows the titration series for SRY HMG-box domain protein. The bottom gel shows cold competition with unlabeled dsDNA demonstrating strength and specificity of binding. The average KD ∼12 nM is close to previously reported values. (B-C) The mouse MAEL HMG-box does not bind to 25-nt ssDNA or dsDNA modified with symmetrical cytosine methylation. (D) Predicted (Robetta) tertiary structure of Drosophila melanogaster (Dm) Mael HMG-box domain. Based on the predicted structure, this domain closely resembles canonical HMG-box domains without any apparent novel regions. Despite the homology and contrary to previous observations dsDNA [27], we did not detect binding to dsDNA. (E) Binding of mouse MAEL HMG-box domain to DNA 4WJ. Top two gels show titration of protein with different concentration ranges. Even at high excess only single MAEL HMG-box domain is able to bind to DNA 4WJ. The bottom gel represents cold competition with unlabeled DNA 4WJ. The binding of the MAEL HMG-box domain is strong: average KD ∼14 nM. (TIF) [file pone.0120268.s003.tif]

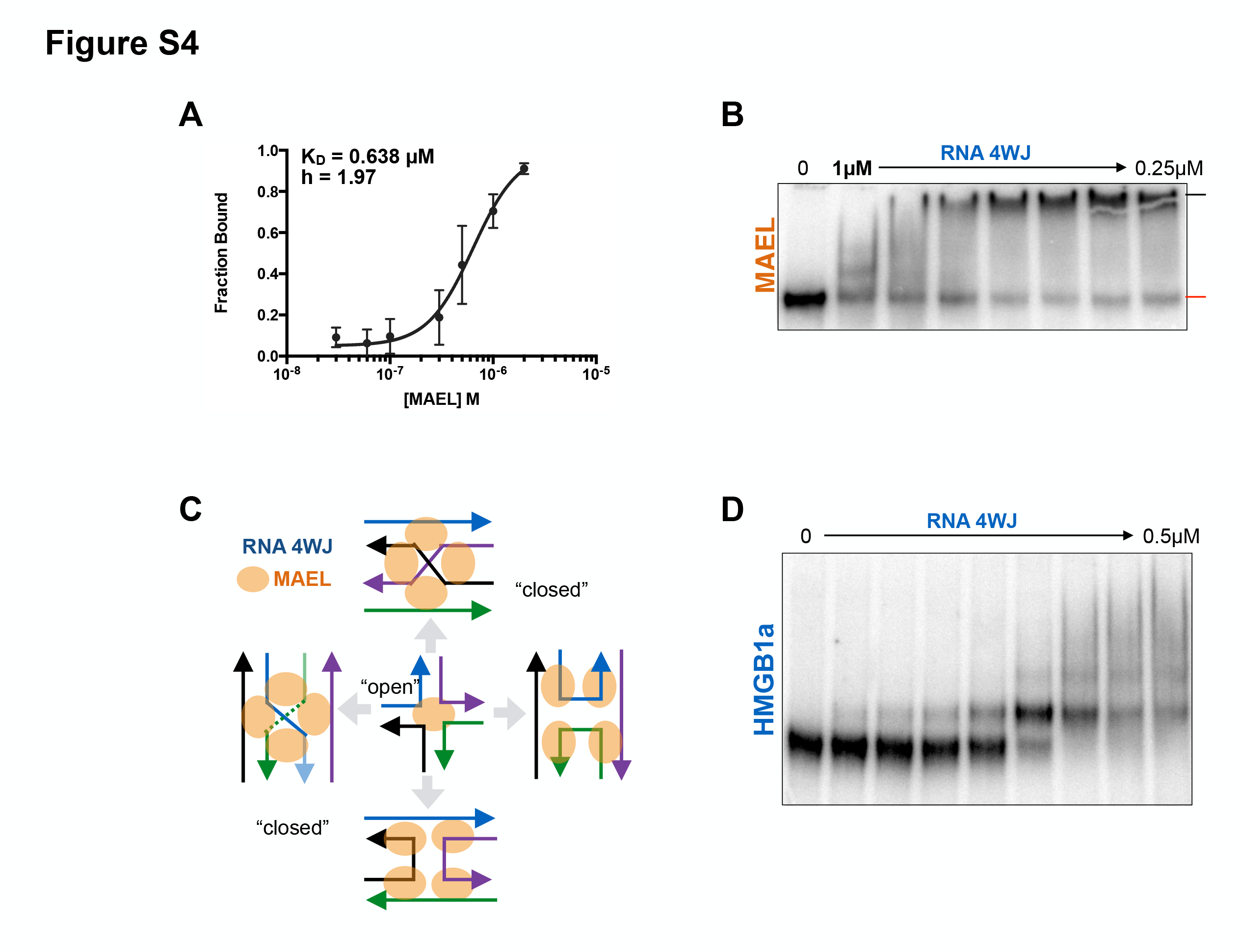

Supplement: S4 Fig — (A) Strength of MAEL HMG-box domain interaction with RNA 4WJ is moderate (KD = 0.638 μM). (B) Cold competition of MAEL HMG-box domain with RNA 4WJ leads to increased formation of the large complex instead of competition. Presence of more RNA likely influences structural ensemble, making the formation of large complex more favorable. (C) Model summarizing structural transition of RNA 4WJ tested here based on previous analysis [80]. The model shows that RNA 4WJs transitions between five conformations—four "closed" and one "open.". The observed small complex likely corresponds to binding to open conformation while the large complex to closed ones. (D) HMGB1a binds well to RNA 4WJ, however, instead of forming single large complex like MAEL HMG-box, it binds progressively, forming multiple intermediate complexes. (TIF) [file pone.0120268.s004.tif]

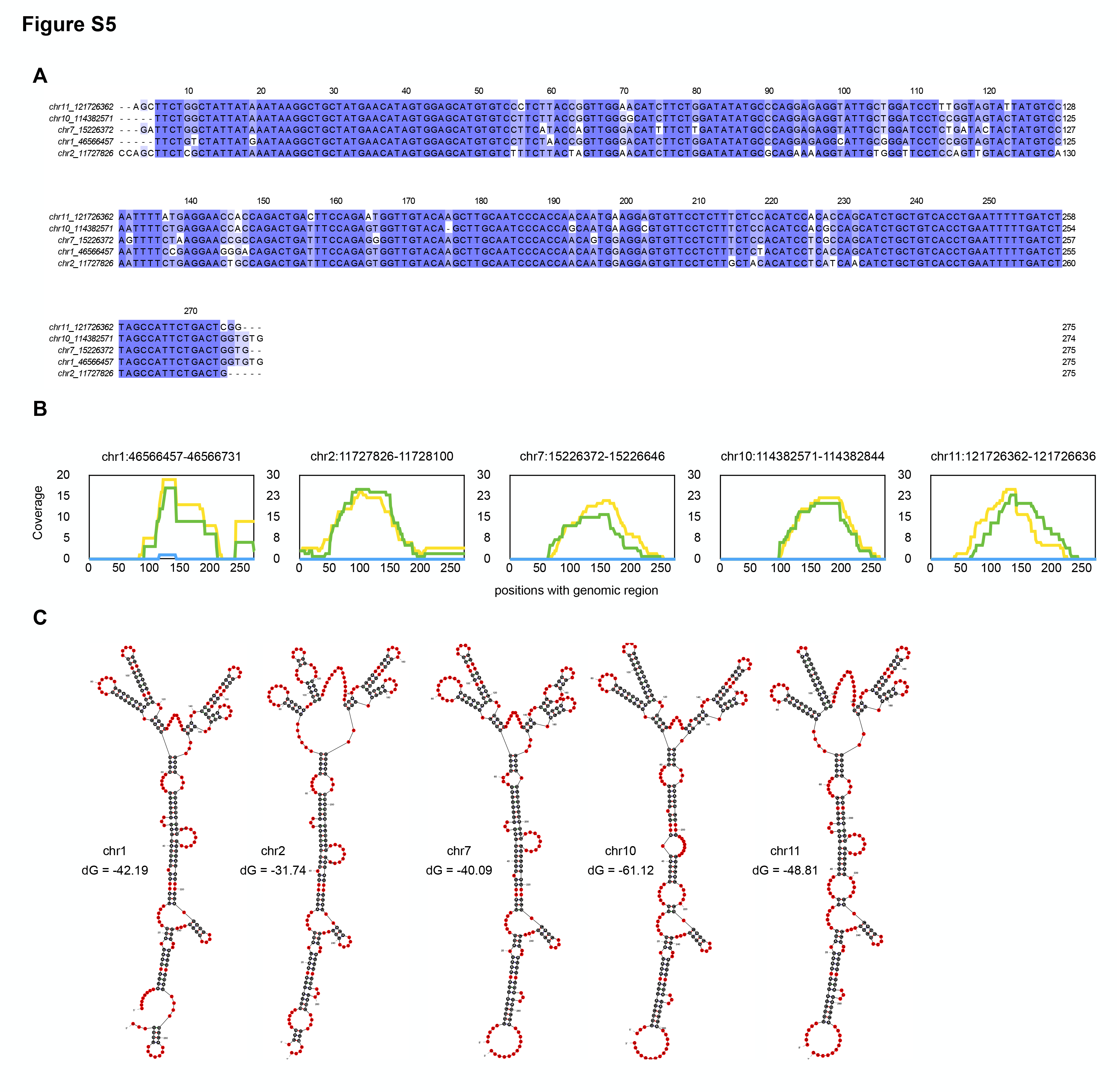

Supplement: S5 Fig — (A) Multiple sequence alignment of regions identified through analysis of MAEL RIP-Seq datasets. Sequences correspond to L1_Md_F2 retrotransposon, are located on different chromosomes, and are highly conserved. Colored by percent identity (JalView). (B) Traces for individual regions after manual refinement of the alignment showing genomic locations and corresponding read coverage in each examined dataset. (C) Single, thermodynamically stable secondary structures generated in Mfold provided with covariance information for each identified genomic region. Shown are dG values of each structure as calculated by Mfold. (TIF) [file pone.0120268.s005.tif]
